# Genome-Wide Association Study for Belly Traits in Canadian Commercial Crossbred Pigs

**DOI:** 10.3390/ani15091254

**Published:** 2025-04-29

**Authors:** Zohre Mozduri, Graham Plastow, Jack Dekkers, Kerry Houlahan, Robert Kemp, Manuel Juárez

**Affiliations:** 1 Livestock Gentec Centre, Department of Agricultural, Food and Nutritional Science, University of Alberta, Edmonton, AB T6G 2E1, Canada; mozduri@ualberta.ca (Z.M.); plastow@ualberta.ca (G.P.); 2 Department of Animal Science, Iowa State University, Ames, IA 50011, USA; jdekkers@iastate.edu; 3 Genesus Genetic Technology Inc., Winnipeg, MB R3P 0H4, Canada; khoulahan@genesus.com (K.H.); bobkemp@genesus.com (R.K.); 4 Lacombe Research and Development Centre, Agriculture and Agri-Food Canada, Lacombe, AB T4L 1W1, Canada

**Keywords:** belly traits, pork quality, QTLs, fat composition, whole-genome sequencing

## Abstract

The improvement of carcass traits is a key focus in pig genetic breeding programs. To identify quantitative trait loci (QTLs) and genes linked to key carcass traits, we conducted a genome-wide association study (GWAS) using whole-genome sequencing data from 1118 commercial pigs (Duroc sires and Yorkshire/Landrace F1 dams). This study focused on six phenotypes: iodine value, belly firmness, belly side fat, total side thickness (belly SThK), belly subcutaneous fat (Subq), and belly seam. Phenotypes were measured using image analysis, DEXA, and fatty acid profiling, and genotyping was performed using low-pass sequencing (SkimSeq). After quality control, 18,911,793 single nucleotide polymorphisms (SNPs) were retained for further analysis. A GWAS was conducted using a linear mixed model implemented in GCTA. Key findings include a significant QTL on SSC15 (110.83–112.23 Mb), which is associated with the iodine value, containing genes such as *COX15*, *CHUK*, *SCD*, and *HIF1AN*, which have known roles in fatty acid metabolism. Additionally, *PNKD*, *VIL1*, and *PRKAG3* (120.74–121.88 Mb on SSC15) were linked to belly firmness, influencing muscle structure and fat composition. Three QTLs for belly side fat were identified on SSC1, SSC2, and SSC3, highlighting genes like *SLC22A18*, *PHLDA2*, and *OSBPL5*, which regulate fat deposition and lipid metabolism. The results provide novel molecular markers that can be incorporated into selective breeding programs to improve pork quality, fat distribution, and meat composition. These findings enhance our understanding of the genetic mechanisms underlying carcass belly traits while offering tools to improve pork quality, optimize fat composition, and align with consumer preferences in the meat production industry.

## Simple Summary

Belly fat traits are key indicators of pork quality and consumer acceptance. In this study, we analyzed 1118 commercial crossbred pigs to identify genetic regions and candidate genes associated with six important belly traits: iodine value, firmness, side fat, total thickness, subcutaneous fat, and seam fat. Using whole-genome sequencing data and genome-wide association studies (GWASs), we discovered several quantitative trait loci (QTLs) and genes involved in fat metabolism, muscle structure, and energy regulation. Genes such as *SCD*, *CHUK*, *HIF1AN*, and *PRKAG3* were highlighted for their contributions to fat composition and meat quality. These findings offer valuable genetic markers for improving pork quality through breeding and provide insights into the biological processes underlying carcass fat traits in pigs.

## 1. Introduction

The pork belly comprises a combination of various component muscles along with layers of intermuscular and subcutaneous fat [1]. Backfat depth is a critical metric for carcass production and an important indicator of pig performance and lean meat yield [2]. Currently, pork belly stands out as the most valuable ($/cwt) retail cut of pork [3]. Pork belly is extracted from the area between the second and third ribs to just above the hip bone and is known for its high fat content (30–60%), though this proportion has decreased over the years [4]. In North America and other regions, pork belly is primarily used for bacon production [5], while various other preparations of raw bellies are preferred in Asian markets [6]. A growing demand for bacon has significantly increased the demand for pork bellies [7]. Increasing demand for pork in Asian and European markets has driven the need for high-quality, specifically defined fresh pork bellies [6]. As a result, it is important to predict pork belly composition and understand the factors that influence it to satisfy market demands [8]. Firmness is a critical characteristic in determining the quality of pork belly for both applications, and excessive softness is considered a significant defect [5].

The fat in pork belly is composed of two layers: subcutaneous and intermuscular fat [1,4]. Excess subcutaneous fat can lead to a greasy taste, making the pork belly less appealing [9]. It has been reported that pork bellies need a minimum level of subcutaneous fat and a higher degree of fat saturation to meet the firmness standards demanded by buyers and processors [10]. While iodine value (IV) is a good indication of the degree of unsaturation in fat and is often used to predict fat firmness, it has been widely reported that IV does not explain more than one-third of the variability in belly firmness [7].

Kang et al. (2023) reported heritability estimates for detailed belly traits, which ranged from moderate to high (0.27 to 0.49) [11]. The genetic correlation among various pork belly parameters, such as the fat ratio, the intermuscular fat area, and the subcutaneous fat area, has been reported to range from −0.24 to 0.84 [12]. Additionally, Do et al. (2014) found moderate to high genetic correlations between carcass traits, with belly weight showing a correlation of 0.88 with carcass weight, 0.46 with back fat thickness, and 0.80 with lean meat percentage [13]. In a commercial crossbred study, trimmed belly weight was genetically correlated with traits such as weaning weight, average daily gain, back fat thickness, and intramuscular fat (IMF) [14]. These findings suggest that fat-related traits in pigs are genetically highly related [1]. Willson et al. (2020) reported that the heritability of belly firmness was 0.31 ± 0.11. The high estimate for belly firmness indicates the potential for its selection in breeding programs [15].

Several studies have identified potential candidate genes linked to backfat and IMF in pigs. For instance, the *DHCR7* gene, located on SSC2 near *IGF2*, has been linked to backfat thickness in multiple pig populations. The *MC4R* polymorphism has been reported to influence backfat thickness, feed intake, and growth traits, including test daily gain and lifetime daily gain [16,17]. However, research on genes related to the component muscles of pork belly is still lacking, highlighting the need for further genetic analysis, including GWAS and -omics studies. This study aimed to determine whether WGS could identify new regions associated with carcass belly fat traits in Canadian commercial crossbred pigs.

## 2. Materials and Methods

### 2.1. Animals and Phenotypes

The experimental procedures were approved by the Animal Care Committee at the Agriculture and Agri-Food Canada Lacombe Research and Development Centre (AAFC-LRDC), under protocol #202204 (June 2024), adhering to the Canadian Council on Animal Care’s guidelines. A total of 1118 commercial crossbred pigs (498 females and 620 males; from Yorkshire × Landrace sows × Duroc boars; Genesus Genetic Technology, London, ON, Canada) were raised according to standard commercial practices and slaughtered at ~125 kg at the AAFC-LRDC federally inspected abattoir. Blood samples were collected for genomic analysis during bleeding (vacutainer tubes, K2 EDTA, 10 mL volume; BD Vacutainer^®^; Mississauga, ON, Canada). Carcass weight and backfat depth, measured between the 3rd and 4th last ribs, were recorded using a grading probe. The carcasses were then divided into primal cuts, and the fat content was determined using dual energy x-ray absorptiometry (DEXA) [18]. A pork chop from the loin center was used to measure the IMF content with the Smart Trac Fat Analyzer Model 907,955 (CEM Corporation, Matthews, NC, USA). A 5 g backfat sample from the shoulder was collected, stored at −80 °C, and analyzed for fatty acids following [19]. Fatty acid composition was used to calculate the IV, according to the American Oil Chemists Society [20]. Images of bellies were taken for image analysis, with measurements including side fat, total side (SThK), subcutaneous fat (Subq), and seam fat (Seam) thicknesses, as well as belly firmness assessed according to previously published protocols [10,21,22].

### 2.2. Genotypes

Blood samples were genotyped for 36,566,734 single nucleotide polymorphism (SNP) markers using SkimSEEK™ (a low-pass sequencing method imputed up to a whole-genome sequence; Neogen^®^, Edmonton, AB, Canada). Quality control procedures were applied to exclude SNPs from the whole-genome sequencing data using PLINK 2.00a3.6 [23], with the following criteria: minor allele frequency (MAF) < 0.01, genotyping rate < 0.01, sample genotyping rate < 0.1 (mind), and Hardy–Weinberg equilibrium (HWE) *p*-value < 1 × 10^−6^. Only autosomal SNPs were included in the analysis. No animals or variants were excluded due to missing genotype data, so imputation was not required. After quality control, 1118 pigs and 18,911,793 SNPs were retained for further analysis.

### 2.3. Statistical Analysis

A linear mixed model was used to assess both fixed and random effects using the lme4 package (version 1.1–35) in the R software. The package was accessed from https://cran.r-project.org/web/packages/lme4/, accessed on 3 November 2023, employing REML or maximum likelihood estimation. The fixed effects tested included sex, boar line, boar group (or semen pool effect), and other methodological factors depending on the trait. The animal model accounted for random additive polygenic effects, as well as random effects for the contemporary group and the common litter. Commercial carcass weight was included as a covariate, and to adjust for population stratification, PCA was performed, incorporating the top three principal components as covariates. For the genome-wide association study, a single-marker mixed linear association model (MLMA) was applied using GCTA version 1.26.0 [24].

The model is represented by

*y* = 1*µ* + *Xb* + *Zu* + *W*_1_*c*_1_ + *W*_2_*c*_2_ + *e*

where *y* denotes the vector of phenotypes for all (*n*) animals; *µ* is the overall mean; *b* is a vector of (*p*) fixed effects (including the additive effect of SNP, sex, boar line, and boar group); *X* is the incidence matrix of fixed effects (*n* × *p*) linking the records in *y* to the fixed effects in *b* in which SNP genotypes are coded as 0, 1, or 2; *u* is a vector of polygenic random effects; and *Z* is an incidence matrix that relates records to the polygenic effects. *c*_1_ and *c*_2_ are vectors of q levels (*q* × 1) of random effects of the contemporary group and the common litter, and *e* represents a vector of random residual terms (*n* × 1). *W*_1_ and *W*_2_ represent design matrices (*n* × *q*_1_ and *n* × *q*_2_) relating to the records in *y* with the random effects in *c*_1_ and *c*_2_, respectively. It is assumed that *u*~*N*(0, GRM *σ*^2^*_u_*) and *e*~*N*(0, *I σ*^2^*_e_*), where GRM is the genomic relationship matrix and *σ*^2^*_u_* and *σ*^2^*_e_* represent the additive genetic and residual variances, respectively.

To correct for multiple testing, we employed the simple method proposed by [25]. This method determines the effective number of independent tests based on the degree of linkage disequilibrium (LD) among SNPs through principal component analysis. First, a correlation matrix for the SNPs was constructed for each chromosome using the composite LD. Next, eigenvalues were calculated from the principal component analysis of the composite LD matrix. Finally, the effective number of SNPs per chromosome was determined as the number of principal components needed to jointly explain 99% of the variance among the SNPs. Since SNPs located on different chromosomes are anticipated to be in linkage equilibrium within the general population, the total effective number of SNPs (Meff) used in this study was derived by summing the effective number of SNPs for each chromosome. *p*-values from single SNP association tests were further adjusted for multiple comparisons using the Šidák correction based on Meff, calculated as follows: adjusted *p*-value = 1 − (1 − *p*-value)^Meff^ [26]. We utilized the qqman R package (version 0.1.9) [27] to generate Manhattan and Q-Q plots for visualizing the GWAS results. The Manhattan plots were customized to highlight significant associations by setting a genome-wide significance threshold at *p* < 2.62410715 × 10^−7^ and color-coding chromosomes for clarity.

#### Sample Size Justification Based on Power Analysis

To ensure that the sample size (*n* = 1118) was sufficient for detecting significant associations in the GWAS, a Power analysis was conducted in the R software (version 4.3.2) using a threshold of α = 2.624 × 10^−7^, based on the method reported by [25] for multiple testing corrections. This method adjusts for linkage disequilibrium between SNPs and provides a more realistic effective number of independent tests compared to Bonferroni corrections. We assessed the statistical power across different effect sizes (Beta) and minor allele frequencies (MAFs), showing that SNPs with an MAF ≥ 0.2 and an effect size (β) ≥ 0.3 have enough statistical power (≥80%) with 1118 individuals. For rarer SNPs (MAF = 0.05), high power would only be achieved for large effect sizes (β ≥ 0.5).

### 2.4. Post-GWAS Analyses

We used the biomaRt package in R (https://bioconductor.org/packages/biomaRt/, accessed on 15 June 2024) to identify candidate genes linked to SNPs in the relevant regions, as well as neighboring SNPs within 0.5 Mbp upstream and downstream based on the *Sus scrofa* 11.1 reference genome assembly, which was obtained from the NCBI Datasets platform (https://www.ncbi.nlm.nih.gov/datasets/genome/GCF_000003025.6/, accessed on 15 June 2024). Genes located within ±0.5 Mb of the SNP positions were extracted and annotated. The 0.5 Mbp distance was chosen based on findings that the average linkage disequilibrium (LD) in commercial pig lines falls below 0.3 for SNPs that are more than 0.5 Mb apart [28]. Thus, the significant window was defined as 0.5 Mb on either side of the significant SNPs identified in the GWAS that were within 0.5 Mb of each other.

Additionally, the proportion of variance explained by each significant SNP was the amount of genetic variance reduced after adding the significant SNP to the model in GCTA version 1.26.0 [24] divided by the phenotypic variance. Finally, we employed the UpSetR package in R, a customizable tool for data exploration and set visualization, to display the number of common SNPs and overlapping genes shared among the six traits in our GWAS analysis, providing a clear alternative to complex Venn diagrams when working with multiple datasets [29].

Functional enrichment analysis was conducted using the DAVID Functional Annotation Tools (https://davidbioinformatics.nih.gov/tools.jsp, accessed on 25 June 2024) [30], employing the Benjamini–Hochberg procedure to adjust for multiple comparisons and control the false discovery rate (FDR).

## 3. Results and Discussion

### 3.1. Descriptive Statistics and Heritability Estimates

Descriptive statistics for eight belly traits are summarized in Table 1. The IV exhibited the largest sample size (*n* = 1117), with a mean of 60.3 and a standard deviation (SD) of 3.34, indicating moderate variation among individuals. Belly firmness demonstrated the highest variability (*SD* = 26.4), with values ranging from 69.8 to 132.0 across a smaller sample size (*n* = 494). Belly side fat and subcutaneous fat (Subq) displayed similar mean values of 2.50 mm and 1.36 mm, respectively, with relatively low standard deviations, suggesting limited dispersion. Total side thickness (SThK) had a mean of 3.71 mm (SD = 0.58), while intermuscular fat (Seam) showed the lowest mean value (2.12 mm) among all measured fat-related traits. These descriptive results provide a quantitative overview of phenotypic variation in economically relevant belly traits in Canadian commercial crossbred pigs and serve as a basis for subsequent genetic analyses.

### 3.2. Iodine Value

**Figure 1:**
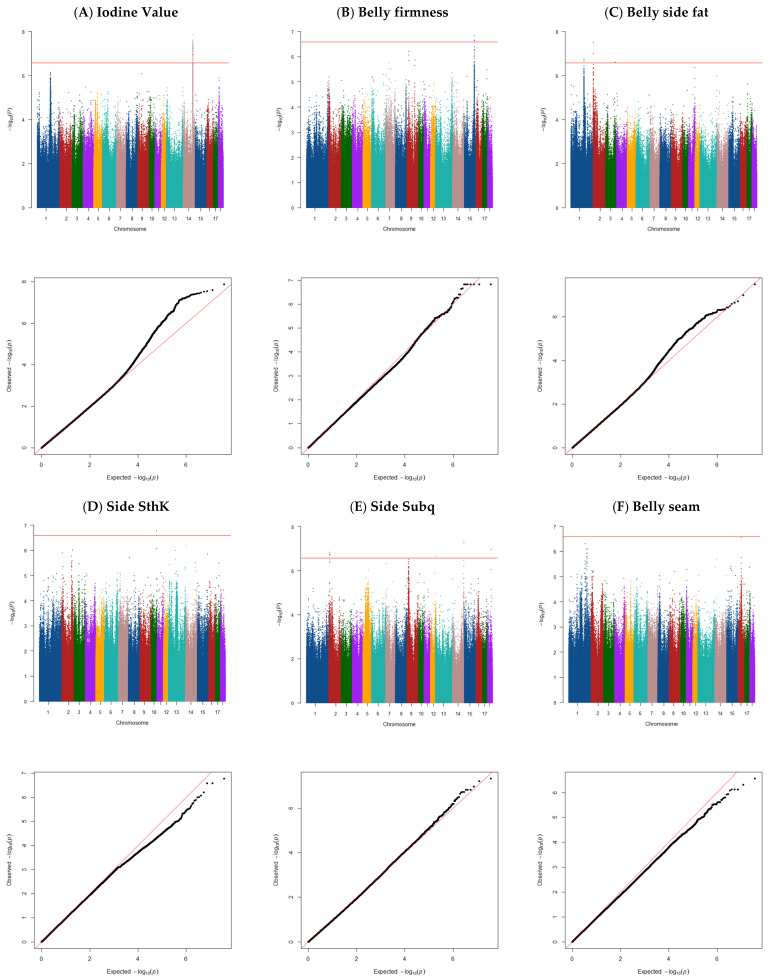
Manhattan and QQ plot of the GWAS for belly traits: (**A**) iodine value (λ = 0.97), (**B**) belly firmness (λ = 0.98), (**C**) belly side fat (λ = 0.96), (**D**) side SthK (λ = 0.96), (**E**) side Subq (λ = 0.94), and (**F**) belly seam (**λ = 0.96**) in commercial pigs. The horizontal red line indicates the genome-wide significance threshold (a significant threshold of *p* < 2.62410715 × 10^−7^, corrected for multiple testing). We applied the corrected threshold for multiple testing using the simple method described by Gao et al. (2008) [25].

The genome-wide association analysis for IV identified one QTL located at 110.83–112.23 Mb on SSC14 (Figure 1A). This QTL explained ~6% of the total variance, and 63 SNPs were significant for IV (Table 2), and 39 genes were found in this region (Table 2 and Supplementary Material S1). Several of the genes located in this QTL region are involved in overlapping pathways regulating fatty acid composition and metabolism. *COX15* was previously found to be associated with fatty acid composition traits, particularly C18:1n-9 and C18:0 [31].

In addition, *CPN1* influences saturated fatty acids (SFAs), with a key SNP identified within its intron [32]; *CHUK* plays a regulatory role in lipid and carbohydrate metabolism, impacting IMF deposition in pigs [33]; both *BLOC1S2* and *SCD* are linked to unsaturated fatty acid content in porcine muscle [34]. The ***SCD*** gene plays a central role in lipid metabolism by catalyzing the conversion of saturated fatty acids (e.g., stearic acid, C18:0) into monounsaturated fatty acids (e.g., oleic acid, C18:1n-9) [35]. Polymorphisms in ***SCD*** have been significantly associated with variation in fatty acid composition in pigs, particularly influencing the ratio of saturated to unsaturated fatty acids [36]. In pigs, genome-wide association studies have identified ***SCD*** as a major candidate gene within QTL regions, affecting intramuscular fat quality and composition [37]. Its proximity to top associated markers and its functional role in fatty acid desaturation highlight ***SCD*** as a valuable target for improving meat quality traits through genomic selection [35]. *PKD2L1* regulates the synthesis of fatty acids, particularly C16:0 and C16:1n-7 [32]. Additional genes such as *WNT8B* influence growth traits in pigs [38], and *NDUFB8* contributes to fatty acid oxidation by encoding a mitochondrial NADH dehydrogenase subunit [39]. *HIF1AN* interacts with multiple genes involved in fatty acid metabolism, playing a central role in regulating fatty acid levels [40]. The *HIF1AN* gene, identified through expression-based genome-wide association studies, is implicated in regulating fatty acid metabolism in pigs. It was significantly associated with 241 SNPs across multiple chromosomes [41]. Its expression may be inhibited through the PI3K-Akt pathway, linking ***HIF1AN*** to lipid regulation and energy metabolism and supporting its role as a candidate gene for intramuscular fatty acid composition [41]. Furthermore, *PAX2* and *SEMA4G* are associated with the synthesis of monounsaturated fatty acids (MUFAs) and specific fatty acids like C16:1n-7 [32]. Lastly, *LZTS2* has been linked to milk fat and protein formation in other species, indicating its broader role in lipid metabolism [42].

The QTL identified for IV highlights key genes regulating fatty acid metabolism and composition, offering potential targets for genetic selection. Genes such as *CHUK*, *SCD*, and *HIF1AN*, which influence lipid synthesis, oxidation, and unsaturated fatty acid content, provide insights into pathways that may be modified to help optimize pork fat quality. Associations with these gene variants may allow breeders to use genomic selection to enhance traits like fat firmness and oxidative stability, which are crucial for meat processing and consumer preferences. The association of multiple genes within overlapping pathways emphasizes the interconnected regulation of fatty acid traits and may help with more precise selection strategies to balance fat quality, composition, and overall meat performance.

### 3.3. Belly Firmness

Genome-wide association analysis for belly firmness identified one QTL (Figure 1B) located at 120.74–121.88 Mb on SSC15 that explained ~1% of the total variance, with nine SNPs significantly linked to the trait (Table 2). Within 0.5 Mb upstream and downstream of these significant SNPs, 69 genes (Table 2 and Supplementary Material S1) were identified. These genes contribute to pathways associated with muscle structure, metabolism, and fatty acid composition, which are critical to meat quality traits. The *PNKD* and *VIL1* genes regulate muscle structural integrity, impacting muscle composition and meat color through variations in myoglobin concentration and metabolism [43,44]. *VIL1* has been linked to meat pH, color, and tenderness in pigs and cattle [45,46,47].

Other studies have shown that the ***PNKD*** gene is implicated in the regulation of muscle development and pork quality traits in pigs [48]. Intronic variants have been associated with feed intake, water holding capacity, loin weight, meat texture, and color, particularly in breeds such as Puławska and Polish Landrace. ***PNKD*** is also predicted to interact with proteins involved in muscle growth and structure, indicating its potential role in muscle physiology [48]. These findings suggest that ***PNKD*** is a promising candidate gene for genetic selection to improve carcass traits and meat quality in pigs. Additionally, *VIL1* plays a key role in cytoskeletal dynamics and villin-mediated actin remodeling, which further supports its influence on muscle quality traits [43].

*PRKAG3*, encoding the γ3 subunit of AMP-activated protein kinase (AMPK), plays a central role in energy metabolism in skeletal muscle and is associated with traits like glycolytic potential and IMF content in breeds such as Large White, Duroc, and Pietrain [49].

The ***PRKAG3*** gene is considered a strong candidate, as it influences meat pH and overall pork quality. Genome-wide analyses in Finnish Yorkshire pigs identified SNPs near ***PRKAG3*** as significantly associated with meat pH [50]. In the study by Verardo et al. (2012), post-GWAS network analysis identified *PRKAG3* as one of the key candidate genes potentially involved in regulating meat pH and muscle cell homeostasis, supporting its relevance to pork meat quality traits [50]. These findings support its potential role in determining meat quality traits.

*CTDSP1* and *miR-26* influence milk fatty acid synthesis and adipocyte development, highlighting their potential roles in fat-related pathways. *WNT6* is associated with fat metabolism in pigs [33]. *WNT6* inhibits adipogenesis by activating the Wnt/β-catenin pathway, which blocks fat cell differentiation through β-catenin stabilization [51].

*SLC23A3* regulates fatty acid composition in meat [52]. ***SLC23A3*** is a key gene involved in regulating fatty acid composition in muscle and adipose tissue [52]. It may influence the levels of polyunsaturated fatty acids (PUFAs) by affecting fatty acid metabolism. A higher expression of ***SLC23A3*** has been associated with increased PUFA content, suggesting a potential role in enhancing meat quality and nutritional value [52]. While its function is better characterized in chicken studies [52], the gene shows promise as a candidate for genomic selection to improve fatty acid traits in livestock.

Genes like *ATG9A*, *GLB1L*, and *OBSL1* are expressed in skeletal muscle and are linked to pig meat quality traits [46].

The identified QTL and associated genes highlight pathways critical to belly firmness, including muscle structure, energy metabolism, and fatty acid composition. Genes such as *PNKD*, *VIL1*, and *PRKAG3* emphasize the interplay between muscle integrity, IMF, and meat color, providing actionable targets for genetic selection. These insights enable breeders to optimize meat quality traits like pH, tenderness, and firmness while aligning fat deposition with processing and consumer preferences. Furthermore, genes involved in fatty acid synthesis, such as *CTDSP1*, *WNT6*, and *SLC23A3*, offer opportunities to fine-tune fat composition, enhancing sensory attributes and market value.

### 3.4. Belly Side Fat Thickness

Three QTLs were identified for belly side fat (Figure 1C). The region on SSC1 (159.49–160.73 Mb) explained 14% of the total variance and was confirmed by two SNPs. Additional windows on SSC2 (1.45–2.45 Mb) and SSC3 (111.96–112.96 Mb) explained 14% and 8% of the variance, respectively, confirmed by two and one SNPs. In total, 78 genes were located within 0.5 Mb upstream and downstream of the significant SNPs in these regions (Table 2 and Supplementary Material S1). Key genes involved in lipid metabolism include *SLC22A18*, which regulates lipid metabolic pathways and shows methylation variations between pig breeds [53], and *SLC22A18* is an imprinted gene linked to fat accumulation [54]. It has been reported that the ***SLC22A18*** gene is involved in lipid metabolism, as its knockdown in mice has been shown to decrease lipid accumulation in the liver, indicating its role in promoting lipid storage [55]. ***SLC22A18*** is associated with lipid metabolism and fat deposition in pigs [56]. Located within QTL regions linked to backfat thickness and feed conversion ratio, this imprinted gene may influence fat accumulation through its regulatory effects on hepatic lipid storage [56]. Its involvement in these economically important traits highlights ***SLC22A18*** as a promising candidate for genetic selection in swine breeding programs.

*PHLDA2* influences glycogen metabolism and fat storage [57]. *OSBPL5*, associated with cholesterol balance, contributes to body composition and fat deposition traits [58]. *NADSYN1*, located within QTL regions alongside *INS* and *IGF2*, plays a critical role in lipid metabolism and glucose regulation [59], while *DHCR7*, linked to backfat thickness, regulates fat deposition [60,61]. *ABHD1* encodes lysolipid lipase, supporting lipid droplet formation and adipose tissue development [62]. *HADHA* and *HADHB* are key players in fatty acid oxidation, impacting fat metabolism and muscle texture [63].

In the skeletal muscle tissue of Landrace weanling pigs, ***HADHA*** was found to be downregulated in individuals with higher growth rates [64]. This downregulation may indicate reduced oxidative metabolic activity, which has been associated with increased intramuscular fat content and a decreased growth rate [65]. Furthermore, HADHA expression has previously been reported to be higher in pig breeds characterized by lower growth rates [66,67,68], supporting its involvement in energy metabolism related to muscle development [64]. Collectively, these findings suggest that HADHA is a key gene exhibiting growth rate-associated expression changes in skeletal muscle and may represent a valuable molecular marker for genetic selection and breeding strategies aimed at improving growth performance in pigs.

Other notable genes include *SSC-miR-10383*, which modulates fat deposition by regulating mRNA networks and is differentially expressed in fat- and lean-type pigs [69]. *EMILIN1* contributes to adipogenesis and growth traits, influencing fat distribution [70], and *KCNK3*, involved in potassium–sodium pump regulation, plays a role in meat tenderness [71]. *KCNK3* has been identified as a molecular marker associated with porcine BAT/beige adipocytes, highlighting its potential involvement in the regulation of thermogenic adipogenesis in pigs [72].

RAB10 facilitates lipophagy, with its silencing leading to increased fat accumulation, underscoring its role in fat degradation [73].

The identified QTLs and associated genes reveal critical pathways regulating fat deposition, lipid metabolism, and related traits in pigs. Genes such as *SLC22A18*, *PHLDA2*, and *NADSYN1* provide actionable targets for genetic selection to optimize fat storage and composition. Fat oxidation regulators like *HADHA* and *HADHB* offer insights into balancing fat metabolism with muscle texture and quality. Furthermore, genes like *EMILIN1*, *SSC-miR-10383*, and *KCNK3* emphasize the interplay between fat distribution, meat quality, and growth traits.

### 3.5. Total Side Thickness

Genome-wide association analysis for SThK identified one QTL (Figure 1D) located at 61.08–62.08 Mb on SSC10 that explained ~10% of the total variance. Three SNPs were significantly linked to SThK (Table 2), and 31 genes were identified in this region (Table 2 and Supplementary Material S1).

### 3.6. Side Subcutaneous Thickness

A total of four QTLs were detected for Subq (Figure 1E). The window on SSC2 (8.08–9.24 Mb) explained 8% of the total variance and was confirmed by 10 SNPs. The window on SSC13 (4.53–5.53 Mb) explained 11% of the total variance and was confirmed by one SNP. Additionally, the window on SSC14 (132.50–133.50 Mb) explained 13% of the total variance and was confirmed by two SNPs.

The other window on SSC18 (44.58–45.58 Mb) explained 10% of the total variance and was confirmed by one SNP (Table 2 and Supplementary Material S1), and 125 genes were identified in this region. Amongst these genes, several are known to influence fat deposition and metabolism, including *MACROD1*, an obesity risk gene associated with BMI regulation [74], and *NAA40*, which is involved in lipid metabolism and fatty acid transport, processes that are essential for energy homeostasis [74]. Additionally, *NAA40* was identified as a novel candidate gene associated with the lipid metabolic process in pigs. Its involvement suggests a potential regulatory role in fatty acid metabolism and muscle lipid composition, contributing to variations in intramuscular fat content [75].

*MARK2*, which is linked to adiposity, impacts fat accumulation and backfat thickness traits [76,77]. *PLAAT3*, which is highly expressed in fatty pig breeds, and *LGALS12*, which regulates fat deposition through adipogenesis, highlight critical pathways for subcutaneous fat accumulation [78,79].

The *SLC22A6* gene plays a role in fatty acid oxidation [74], while *BSCL2*, which encodes seipin, regulates fat storage and meat quality traits such as loin mass and carcass yield [80]. It has been reported that the *BSCL2* gene was identified as one of the key genes involved in lipid droplet formation in pigs [81]. The research showed that *BSCL2* expression was associated with adipocyte size across different fat depots (subcutaneous, visceral, and intramuscular), suggesting its potential role in regulating fat accumulation and adipose tissue development [81].

*GANAB* influences protein glycosylation, which affects biological processes related to protein stability and function [82]. *TUT1* has been linked to fatty acid composition and sensory tenderness in meat [83]. The *HOXA* gene family, including *HOXA1*, *HOXA5*, *HOXA9*, and *HOXA13*, plays significant roles in musculoskeletal system development, fat metabolism, and subcutaneous fat deposition. For example, *HOXA5* suppresses adipocyte proliferation and is linked to fat distribution and adipose differentiation in pigs and other species [84,85]. It has been reported that *HOXA10*, *HOXA9*, and *HOXA7* play a role in lipid metabolism in Duroc pigs [34].

The identified QTLs and associated genes reveal a complex network regulating subcutaneous fat deposition, lipid metabolism, and fat distribution. Genes like *MACROD1*, *NAA40*, and *BSCL2* provide actionable targets for genetic selection to optimize fat storage and improve carcass composition. The role of the *HOXA* gene family underscores the genetic regulation of subcutaneous fat and its influence on meat quality traits such as texture and fat distribution.

### 3.7. Belly Seam Fat Thickness

For the belly seam trait, no significant peaks were observed (Figure 1F).

Functional enrichment analysis was performed separately for the gene sets associated with each of the belly traits evaluated in this study. No significantly enriched biological pathways were identified after multiple testing corrections, as the majority of FDR-adjusted *p*-values were equal to 1.0. These findings indicate a lack of overrepresented functional categories among the genes associated with the six belly traits.

### 3.8. Common SNPs and Genes in Windows Associated with Multiple Traits

**Figure 2:**
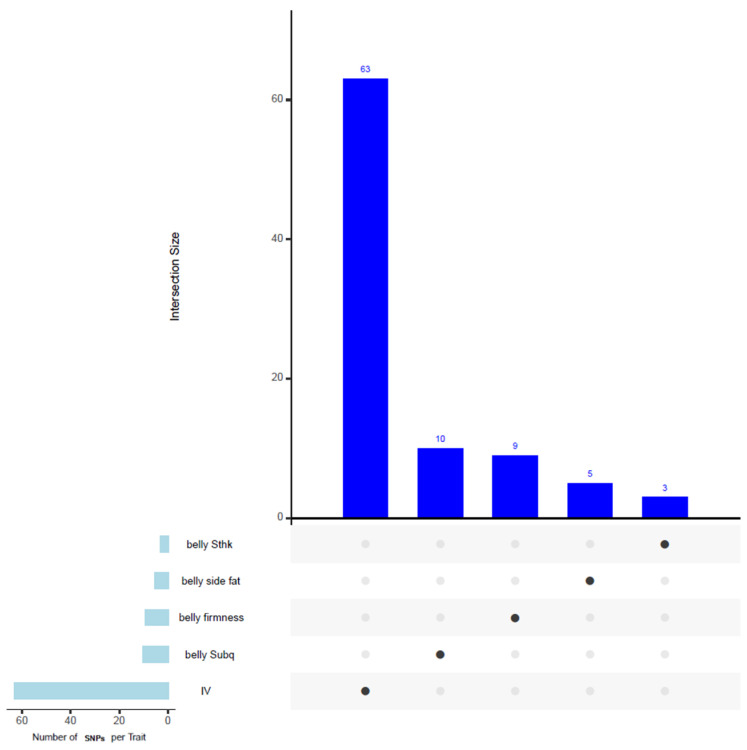
Upset plot illustrating the overlapping SNPs associated with multiple traits identified in the GWAS analysis. Although several SNPs were detected for each individual trait, no shared SNPs were observed across the traits shown. The full list of identified SNPs is provided in Supplementary File S2.

Figure 2 presents an upset plot illustrating the overlapping SNPs among traits in our GWAS analysis. However, no shared SNPs were identified among the traits shown in the figure.

**Figure 3:**
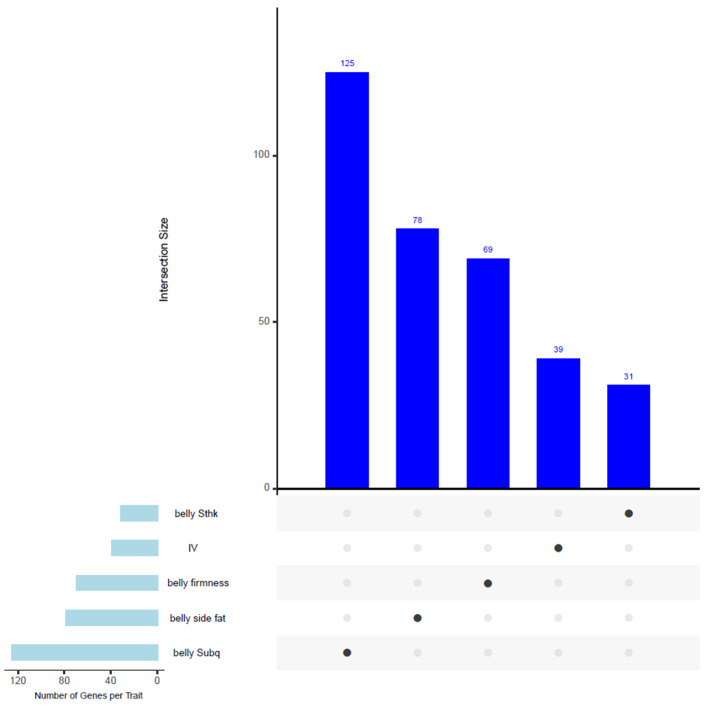
Upset plot showing the overlapping genes located within associated windows across multiple traits in the GWAS analysis. Despite detecting candidate genes for each trait, no common genes were identified among the traits displayed in this figure.

All the SNPs are included in Supplementary File S2. Figure 3 illustrates an upset plot showing the overlapping genes shared among traits in our GWAS analysis. However, no common genes were identified among the traits presented in this figure.

## 4. Conclusions

This study provides valuable insights into the genetic basis of carcass belly traits in pigs using GWAS analysis with whole-genome sequencing data. By analyzing 1118 commercial crossbred pigs from three breeds, we identified significant QTLs and candidate genes associated with traits such as belly seam fat, subcutaneous belly fat, belly firmness, IV, and belly side fat. A larger population can be used in the future to confirm these results and to evaluate rarer SNPs.

This study identifies key genetic markers and pathways influencing carcass belly traits in pigs through GWAS analysis with whole-genome sequencing data. Several QTLs and candidate genes were associated with belly firmness, belly side fat, total side thickness, subcutaneous belly fat, and IV. Genes involved in lipid metabolism, fat deposition, and muscle structure were highlighted, offering actionable targets for genomic selection programs. Integrating these findings into breeding strategies can optimize carcass composition, improve meat quality, and support industry efforts to balance fat content and processing efficiency. This study provides a genetic framework for developing high-quality pork products tailored to consumer and market demands.

## Figures and Tables

**Table 1:** Descriptive statistics for belly traits in Canadian commercial crossbred pigs.

Trait	n	min	max	mean	SD
IV	1117	50.9	75.7	60.3	3.34
Belly firmness (°)	494	69.8	177	132	26.4
Belly side fat (mm)	1083	0.95	4.00	2.50	0.45
Belly SThK (mm)	964	2.00	5.49	3.71	0.58
Belly Subq (mm)	1083	0.82	2.50	1.36	0.23
Belly Seam (mm)	964	0.40	4.07	2.12	0.58

IV: iodine value; SThK: total side thicknesses; Subq: subcutaneous fat; Seam: intermuscular fat.

**Table 2:** Candidate genes located on significant regions and/or nearby regions identified using a whole-genome sequence (WGS) for belly traits in Canadian commercial crossbred pigs.

Trait	Chr	Start, BP	End, BP	SNP (Lowest *p*-Value)	MAF	*p*-Value	Var ^1^	Candidate Genes ^2^
Iodine value	14	110,834,310	112,238,899	14:111437307C:T	0.329	1.33 × 10^−8^	0.06	ENTPD7, COX15, CUTC, ABCC2, Y_RNA, DNMBP, CPN1, CHUK, CWF19L1, SNORA12, BLOC1S2, PKD2L1, SCD, WNT8B, SEC31B, NDUFB8, HIF1AN, ENSSSCG00000044499, ENSSSCG00000049992, ENSSSCG00000058770, PAX2, SLF2, SEMA4G, ssc-mir-10382, MRPL43, TWNK, LZTS2, ENSSSCG00000053081, SFXN3, KAZALD1, and TLX1
Belly firmness	15	120,747,942	121,885,708	15:121247942C:T	0.105	1.49 × 10^−7^	0.01	GPBAR1, AAMP, PNKD, CATIP, SLC11A1, CTDSP1, MIR26B, VIL1, USP37, CNOT9, PLCD4, BCS1L, RNF25, STK36, TTLL4, CYP27A1, PRKAG3, WNT6, WNT10A, CDK5R2, FEV, CRYBA2, ssc-mir-375, CFAP65, IHH, NHEJ1, SLC23A3, CNPPD1, RETREG2, ZFAND2B, ABCB6, ATG9A, ANKZF1, GLB1L, STK16, TUBA4A, DNAJB2, PTPRN, MIR153-1, DNPEP, ssc-mir-4334, DES, GMPPA, ASIC4, CHPF, TMEM198, OBSL1, INHA, STK11IP, SLC4A3, and U6
Belly side fat	1	159,498,384	160,730,075	1:160230075A:C	0.182	1.87 × 10^−7^	0.14	RNF152 and CDH20
Belly side fat	2	1,451,972	2,455,805	2:1951972T:C	0.034	3.19 × 10^−8^	0.14	INS, TH, ASCL2, TSPAN32, CD81, TSSC4, TRPM5, KCNQ1, CDKN1C, SLC22A18, PHLDA2, NAP1L4, CARS1, OSBPL5, NADSYN1, and DHCR7
Belly side fat	3	111,960,719	112,960,719	3:112460719T:C	0.019	2.45 × 10^−7^	0.08	TCF23, PREB, ABHD1, KHK, EMILIN1, OST4, AGBL5, TMEM214, MAPRE3, DPYSL5, CENPA, SLC35F6, KCNK3, CIB4, CIMIP2C, OTOF, DRC1, SELENOI, ADGRF3, HADHB, HADHA, GAREM2, RAB10, and U6
Side SthK	10	61,089,623	62,089,643	10:61589642T:C	0.205	1.68 × 10^−7^	0.1	ENSSSCG00000061765, ENSSSCG00000045932, ENSSSCG00000055980, andENSSSCG00000052436
side Subq	2	8,082,914	9,247,816	2:8587647A:T	0.017	1.47 × 10^−7^	0.08	MACROD1, OTUB1, COX8A, NAA40, RCOR2, MARK2, SPINDOC, ZFTA, ATL3, PLAAT3, LGALS12, PLAAT5, SLC22A8, SLC22A6, SLC3A2, U2, SNORD26, SNORD27, SNORD28, SNORD22, SNORD29, SNORD30, SNORD31, WDR74, TEX54, STX5, NXF1, TMEM223, TMEM179B, TAF6L, POLR2G, TTC9C, HNRNPUL2, BSCL2, UBXN1, UQCC3, CSKMT, SNORA57, C11orf98, INTS5, GANAB, B3GAT3, ROM1, EML3, MTA2, and TUT1
side Subq	13	4,535,728	5,535,728	13:5035728A:G	0.012	2.24 × 10^−7^	0.11	TBC1D5 and U6, SATB1
side Subq	14	132,507,485	133,507,498	14:133007485G:C	0.014	4.65 × 10^−8^	0.13	PSTK, IKZF5, ACADSB, ssc-mir-4331-2, HMX3, HMX2, BUB3, GPR26, CPXM2, and CHST15
side Subq	18	44,588,816	45,588,816	18:45088816A:G	0.014	1.06 × 10^−7^	0.1	JAZF1, TAX1BP1, HIBADH, U6, EVX1, HOXA13, HOXA11, HOXA10, ssc-mir-196b-1, HOXA9, HOXA7, HOXA5, HOXA3, HOXA2, and HOXA1

^1^: all the genes are included in the Supplementary File. ^2^: Var: percentage of additive genetic variance explained by each SNP.

## Data Availability

Data may be available from the authors upon reasonable request and authorization from the funding organizations.

## Supplementary Materials

The following supporting information can be downloaded at https://www.mdpi.com/article/10.3390/ani15091254/s1. Supplementary Material S1. Candidate genes located in significant or nearby regions identified in this study through whole-genome sequencing (WGS) for belly traits, including iodine value, belly firmness, belly side fat, total side thickness, and belly subcutaneous fat in Canadian commercial crossbred pigs. Supplementary Material S2. List of significant SNPs identified in this study.
